# Supplementary material for: A high-resolution mRNA expression time course of embryonic development in zebrafish
Source: eLife. 2017 Nov 16;6:e30860. doi: 10.7554/eLife.30860 (PMC5690287; doi:10.7554/eLife.30860)
Supplement: Supplementary file 6. [file elife-30860-supp6.zip › biolayout-clusters-files/Cluster216.html]

Cluster216


# Cluster216: Detail

### Go to ZFA detail

## GO

| GO ID | Description | Domain | Annotated | Expected | Observed | Adjusted p-value | Genes | Ensembl IDs |
| --- | --- | --- | --- | --- | --- | --- | --- | --- |
| GO:0006606 | protein import into nucleus | biological\_process | 28 | 0.02 | 2 | 0.025 | nup133 nup205 | ENSDARG00000010078 ENSDARG00000042530 |
| GO:0005643 | nuclear pore | cellular\_component | 23 | 0.01 | 2 | 0.018 | nup133 nup205 | ENSDARG00000010078 ENSDARG00000042530 |
